# The Aminosteroid Derivative RM-133 Shows *In Vitro* and *In Vivo* Antitumor Activity in Human Ovarian and Pancreatic Cancers

**DOI:** 10.1371/journal.pone.0144890

**Published:** 2015-12-14

**Authors:** Lucie Carolle Kenmogne, Diana Ayan, Jenny Roy, René Maltais, Donald Poirier

**Affiliations:** 1 Laboratory of Medicinal Chemistry, Endocrinology and Nephrology Unit, CHU de Québec—Research Center, Québec, Québec, Canada; 2 Department of Molecular Medicine, Faculty of Medicine, Université Laval, Québec, Québec, Canada; Wayne State University School of Medicine, UNITED STATES

## Abstract

Ovarian and pancreatic cancers are two of the most aggressive and lethal cancers, whose management faces only limited therapeutic options. Typically, these tumors spread insidiously accompanied first with atypical symptoms, and usually shift to a drug resistance phenotype with the current pharmaceutical armamentarium. Thus, the development of new drugs acting via a different mechanism of action represents a clear priority. Herein, we are reporting for the first time that the aminosteroid derivative RM-133, developed in our laboratory, displays promising activity on two models of aggressive cancers, namely ovarian (OVCAR-3) and pancreatic (PANC-1) cancers. The IC_50_ value of RM-133 was 0.8 μM and 0.3 μM for OVCAR-3 and PANC-1 cell lines in culture, respectively. Based on pharmacokinetic studies on RM-133 using 11 different vehicles, we selected two main vehicles: aqueous 0.4% methylcellulose:ethanol (92:8) and sunflower oil:ethanol (92:8) for *in vivo* studies. Using subcutaneous injection of RM-133 with the methylcellulose-based vehicle, growth of PANC-1 tumors xenografted to nude mice was inhibited by 63%. Quite interestingly, RM-133 injected subcutaneously with the methylcellulose-based or sunflower-based vehicles reduced OVCAR-3 xenograft growth by 122% and 100%, respectively. After the end of RM-133 treatment using the methylcellulose-based vehicle, OVCAR-3 tumor growth inhibition was maintained for ≥ 1 week. RM-133 was also well tolerated in the whole animal, no apparent sign of toxicity having been detected in the xenograft studies.

## Introduction

As a major public health concern worldwide, cancer is responsible for one in four deaths in the USA and Canada [1,2]. Ovarian and pancreatic cancers are two aggressive cancers that share the characteristics of spreading insidiously while displaying atypical symptoms, and readily shifting to a drug resistance phenotype. Ovarian cancer is a heterogeneous disease that afflicts yearly 225,000 women worldwide [3–5]. It is the most lethal among the gynecologic malignancies, due to its asymptomatic nature in its early etiology, and the lack of efficient diagnostic tools [2,5]. As a result, 75% of women already present themselves with advanced stages of ovarian cancer (stages III- IV of the International Federation of Gynecology and Obstetrics classification) when first diagnosed [3,6–9]. For these women, the 5-year survival rate varies from 25% to 35% [3,10] and the gold standard treatment is surgical debulking and chemotherapy based on a combination of paclitaxel- and platinum-based regimens [6,11–13]. Although initial response to ovarian cancer treatment is favorable, the majority of patients become resistant to currently used treatments and more than 90% are subject to relapses after 18 months [14,15]. On the other hand, pancreatic cancer, which is the fourth most lethal cancer, is an aggressive neoplasm presenting with a very poor prognosis [1,2,16]. Its 5-year survival rate is of only 6% [1] due to late initial diagnosis, rapid progression of disease, and resistance to chemotherapy regimens in current use [17]. As pancreatic tumors are characterized by extensive local invasion and early lymphatic as well as hematogenous metastases [18], only few patients are candidates to resection, and therefore, systemic gemcitabine-based chemotherapy is the most currently used form of treatment [19]. Despite recent improvements in drug development, the length and quality of life of pancreatic cancer patients have not improved [20]. Thus, there is an urgent need to develop new approaches for the management of these diseases, which are among most aggressive and lethal cancers and have limited therapeutic options, especially for patients with unresectable pancreatic cancer, whose general condition rapidly deteriorates [16,20,21].

**Fig 1:**
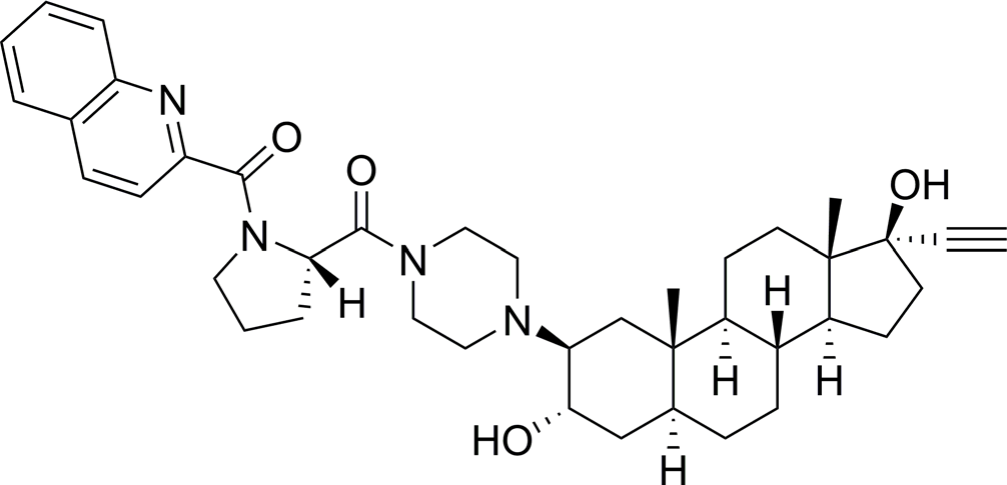
The structure of aminosteroid RM-133.

The above considerations indicate that the development of novel therapeutic agents acting via different mechanisms of action is critically needed to overcome the major problem of drug resistance encountered with these two cancer types, and therefore, to improve the overall survival of patients. Compound RM-133 (Fig 1) is an aminosteroid that has been developed in our laboratory [22]. It has shown antiproliferative activity on several cancer cell lines (HL60 human promyelocytic leukemia cells, T-47D human breast carcinoma cells, WEHI-3 mouse myelomonocytic leukemia cells, and LNCaP human prostate cancer cells) with IC_50_ values ranging from 0.1 to 2 μM [23]. RM-133 also showed a low to moderate risk of drug-drug interactions based on its weak inhibition of the liver enzymes CYP3A4 and CYP2D6 [23]. In a foregoing *in vivo* assay, RM-133 blocked by 57% the growth of HL60 tumor xenografted in nude mice [22]. In a preliminary study of the mechanism of action of this family of aminosteroids, an analog of RM-133 blocked HL60 cells in G0/G1 phase and induced apoptosis [24].

Herein, we report the promising anticancer activity of RM-133 on human adenocarcinoma OVCAR-3 cells, which is a model for drug resistance investigation in ovarian cancer [25], and on human pancreatic carcinoma (PANC-1) cells. We also report pharmacokinetic studies on RM-133 involving various vehicles, as well as its antitumor activity in models of ovarian and pancreatic cancers, namely the OVCAR-3 and PANC-1 tumors xenografted into nude mice.

## Materials and Methods

### Cell lines and cell culture

Human ovarian (OVCAR-3) and pancreatic (PANC-1) cancer cells were purchased from the American Type Culture Collection (ATCC, Rockville, MD) and maintained in exponential growth in a 5% CO_2_ humidified atmosphere at 37°C. OVCAR-3 cells were routinely grown in RPMI-1640 medium (Sigma, St. Louis, MO) supplemented with 20% FBS, L-glutamine (2 mM), antibiotics (100 IU penicillin/mL and 100 μg streptomycin/mL), insulin (50 ng/mL), and estradiol (1 nM). For OVCAR-3 cell proliferation assays, the medium used was identical, except for the omission of estradiol. PANC-1 cells were maintained in DMEM-high glucose (Invitrogen, Burlington, ON, Canada) containing L-glutamine (2 mM) and antibiotics (100 IU penicillin/mL and 100 μg streptomycin/mL), and supplemented with 10% FBS. For PANC-1 cell proliferation assays, the latter maintenance medium was replaced with DMEM/F12 containing the same supplements.

### Cell viability assays

The cell proliferation assay was performed with a colorimetric method using 3-(4,5-dimethylthiazol-2-yl)-5-(3-carboxymethoxyphenyl)2-(4-sulfophenyl)-2H-tetrazolium (MTS) (Cell Titer 96 Aqueous, Promega, Madison, WI). Microtiter 96-well plates (Becton–Dickinson Company, Lincoln Park, NJ) were seeded with 1 × 10^4^ cells/well suspended in 100 μL of medium and pre-incubated for 24 h at 37°C in a 5% CO_2_ atmosphere. A stock solution of RM-133 was prepared in EtOH (1×10^−2^ M) and was diluted into experimental medium, which was added to each well at time zero. After a 72-h incubation, MTS (20 μL of the solution provided by the manufacturer) was added to each well and the plate incubated for 4 h. MTS is converted to water-soluble colored formazan by dehydrogenases present in metabolically active cells, and therefore, the MTS assay allows the immediate determination of absorbance of the soluble formazan directly in the cell medium and thus the measurement of viable cells. The *A*_490_ of the medium was determined using a 96-well microplate reader INFINITE 200 PRO series (TECAN, Männedorf, Switzerland). The IC_50_ value of RM-133 was determined for each of the two cell lines using the GraphPad Prism 7 software (GraphPad Software, Inc., San Diego, CA).

### Animals

All *in vivo* experiments were approved by our Institutional Animal Care and Use Committee (Comités de protection des animaux de l’Université Laval) and carried out according to the guidelines of the Canadian Council on Animal Care. For xenografts, homozygous ♀ *nu/nu* nude mice (24–42 days old) were purchased from Charles River Inc. (Saint-Constant, QC, Canada) and housed (four to five) in vinyl micro-isolated ventilated cages, equipped with air lids, which were kept in laminar airflow hoods and maintained under pathogen-limiting conditions. During the acclimatization and study period, the animals were housed under a controlled environment at 22 ± 3°C, with 50 ± 20% relative humidity and light set at 12 h/day (light on at 07:15). Rodent food (Rodent diet #T.2018.15, Harlan Teklad, Madison, WI) and water were provided *ad libitum*. For *nu/nu* nude mice, food and water were sterilized prior to dispensing to the animals. Standard Balb/c mice were used for pharmacokinetic studies. Animals were anesthetized with isoflurane and killed by cervical dislocation.

### Effect of RM-133 on OVCAR-3 xenografts and drug plasma concentration in nude mice

#### First part

Twenty-two ♀ *nu/nu* nude mice (22–24 g) were inoculated s.c. with 5×10^6^ OVCAR-3 cells (in 0.1 mL of growth medium containing 30% Matrigel (BD Biosciences, Bedford, MA)) into both flanks in each mouse via a 2.5-cm long 22-gauge needle. After 11 days, tumor-bearing mice were randomly assigned to two groups of 11 mice each according to tumor size, i.e. a control group (*n* = 12 tumors) and a treated group (*n* = 15 tumors). RM-133 was administered s.c. daily at 60 mg/kg in 0.1 mL of propylene glycol:EtOH (92:8). Animals in the control group received 0.1 mL of vehicle alone. Tumor size was measured twice weekly using a caliper. Two perpendicular diameters (*L* and *W*) were measured, and the tumor area (in mm^2^) was calculated using the formula (*L*/2) x (*W*/2) x π. Animals were weighed at various intervals during the experiment.

#### Second part

At the end of the xenograft experiment, and in order to obtain preliminary pharmacological data, mice in treated groups were separated into 4 subgroups of 2–3 mice each, injected s.c. with RM-133 (60 mg/kg) and sacrificed after 3, 7, 12, or 24 h. In parallel, mice in the control group were also separated into 4 subgroups, treated with RM-133 (60 mg/kg) as for treated mice, and sacrificed at the same intervals. Blood was collected from mice by cardiac puncture, and the plasma concentration of RM-133 determined by liquid chromatography–tandem mass spectrometry (LC-MS/MS) as previously reported [23]. Tumors of the mice in the treated group were collected at necropsy, pooled, homogenized according to a known procedure [26] and the quantity of RM-133 was determined by LC-MS/MS.

### Plasma concentration of RM-133 in mice at different injection doses

Eight ♀ Balb/c mice weighing approximately 20 g were separated in 4 groups. Mice in each group received a single s.c. injection of RM-133 (30, 60, 120, or 480 mg/kg). The vehicle used was propylene glycol:EtOH (92:8) and injection volume was 0.1 mL for all concentrations except at 480 mg/kg, where 0.2 mL was used. Blood was collected by cardiac puncture after 12 h, and the plasma concentration of RM-133 determined by LC-MS/MS as above.

### Analysis of different vehicles for RM-133 administration

Eleven different vehicles were investigated by s.c. injection [27–35]. These vehicles were formulated as follows: vehicle #1 [propylene glycol:EtOH (92:8)]; vehicle #2 [aqueous 0.4% methylcellulose:EtOH (92:8)]; vehicle #3 [castor oil:EtOH:benzyl alcohol:benzyl benzoate (65:10:10:15)]; vehicle #4 [sunflower oil:EtOH (92:8)]; vehicle #5 [sunflower oil:tetrahydrofuran (92:8)]; vehicle #6 [aqueous 25% β-cyclodextrin:EtOH (92:8)]; vehicle #7 [sesame oil:EtOH:benzyl benzoate:benzyl alcohol:Tween 80 (89.8:7.8:1:1:0.5)]; vehicle #8 [soya oil:EtOH (92:8)]; vehicle #9 [RPMI medium:EtOH (92:8)]; vehicle #10 [saline:dimethyl sulfoxide (DMSO):Tween 80 (89.5:10:0.5)]; and vehicle #11 [saline:benzyl alcohol:carboxymethylcellulose:Tween 80 (98.2:0.9:0.5:0.4)]. The eight vehicles producing the best RM-133 solubility (#1–8) were injected s.c. (0.1 mL) without the test compound (RM-133) for tolerance and behavioral evaluation studies, by observing mice at 0.25, 1, 3, 7, 23, 26, 28, 30, and 96 h post-injection. Finally, RM-133 was injected s.c. once at 120 mg/kg/0.1 mL of the best seven tolerated vehicles (#1,2,4–7) into ♀ Balb/c mice (4 per group), and mice were next monitored with blood collection by cardiac puncture after 3 and 12 h. The plasma concentration of RM-133 was then determined by LC-MS/MS as above.

### Effect of repeated s.c. injections of RM-133 using 3 vehicles

Ten ♀ Balb/c mice weighing approximately 23 g were separated in 3 groups. Group 1 (3 mice) was treated s.c. with RM-133 (240 mg/kg/0.2 mL, twice a day (AM and PM), every other day) in aqueous 0.4% methylcellulose:EtOH (92:8). Group 2 (3 mice) was treated s.c. with RM-133 (120 mg/kg/0.1 mL, twice a day (AM and PM), every other day) in sunflower oil:EtOH (92:8). Group 3 (4 mice) received only one injection of RM-133 (120 mg/kg/0.1 mL) in aqueous 25% β-cyclodextrin:EtOH (92:8) and were sacrificed 24 h later. Mice in groups 1 and 2 received a total of 8 injections over 4 days and were sacrificed on day 8. Mice behavior was monitored during the whole duration of the experiment, and macroscopic observation of organs was performed upon necropsy.

### Effect of RM-133 on OVCAR-3 xenografts using the two selected vehicles

Fourty ♀ *nu/nu* nude mice (22–24 g) were inoculated with 5×10^6^ OVCAR-3 cells per flank, in 30% matrigel-containing culture medium (0.1 mL). After 23 days, tumor-bearing mice were randomized in 4 groups of 8 to 9 mice each. Group 1 (12 tumors) was treated s.c. with RM-133 (240 mg/kg/0.2 mL, once (AM) every other day) in sunflower oil:EtOH (92:8), and group 2 (13 tumors) was treated s.c. with RM-133 (240 mg/kg/0.2 mL, twice a day (AM and PM), every other day) in aqueous 0.4% methylcellulose:EtOH (92:8). Two control mouse groups, each representing 12 tumors, received only the vehicles. As mentioned above, the tumor area was measured twice weekly and body weight monitored.

### Effect of RM-133 on PANC-1 xenografts

Twenty female *nu/nu* nude mice (22–24 g) were inoculated with 5×10^6^ PANC-1 cells per flank, in 30% matrigel-containing culture medium (0.1 mL). Ten days after inoculation, tumor-bearing mice were randomized in 2 groups of 10 mice each. Mice in the first group (*n* = 16 tumors) were treated s.c. with RM-133 (240 mg/kg, twice a day (AM and PM), every other day) in 0.2 mL of aqueous 0.4% methylcellulose:EtOH (92:8). Mice in the second (control) group (*n* = 18 tumors) received the vehicle only. As with previous xenografts, the tumor area was measured and mouse body weight monitored.

### Synthesis and preparation of RM-133

The aminosteroid RM-133 (2β-[1-(quinoline-2-carbonyl)-pyrrolidine-2-carbonyl] *N*-piperazine-5α-androstane-3α,17β-diol) was synthesized, characterized, and purified as previously described [22]. The purity of RM-133 was found to be 99.5% as determined by high performance liquid chromatography (Apparatus: Shimadzu (Kyoto, Japan); Column: Altima, HP, C18-AQ, 4.6 x 250 mm, 5 μm; Solvents: A gradient from methanol/water (70:30) to 100% methanol; UV detection at 190 nm). For *in vivo* treatments, RM-133 was suspended in the vehicle one day prior to its injection into mice and stored at 4°C under constant agitation until use.

### Statistics

The Duncan-Kramer test was used to analyze data, and statistical significance accepted at *P* < 0.05 [36].

## Results and Discussion

### RM-133 is cytotoxic towards human OVCAR-3 and PANC-1 cells

**Fig 2:**
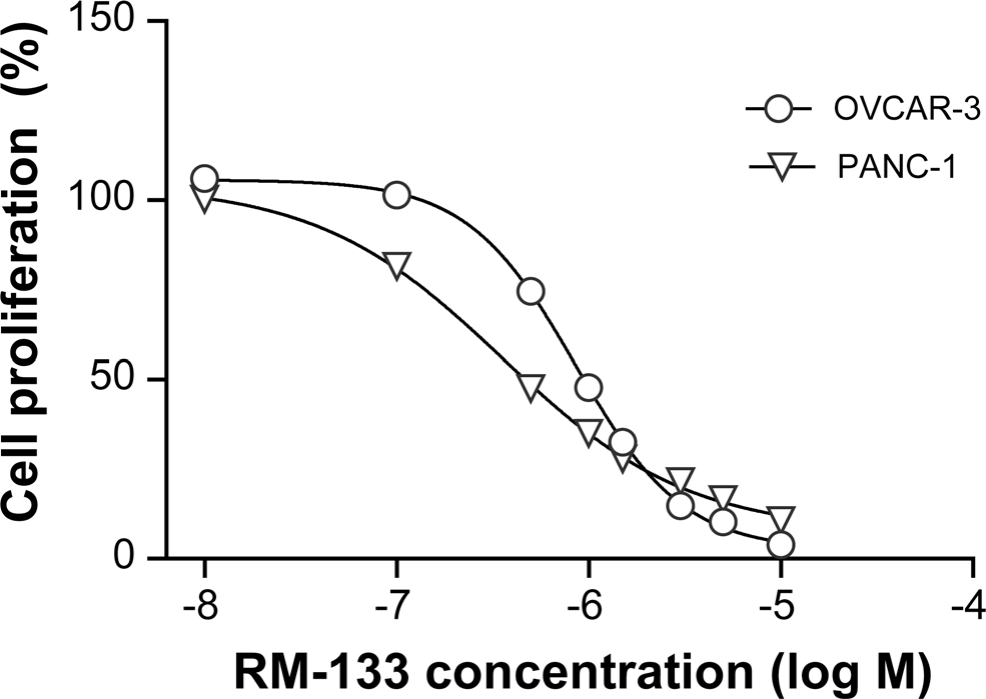
Effect of increasing concentrations of RM-133 on OVCAR-3 and PANC-1 cell growth. The IC_50_ values were calculated as 0.8 μM (OVCAR-3) and 0.3 μM (PANC-1). Data represent the mean ± S.D. The error bars are smaller than the symbols.

To evaluate the effect of RM-133 on the proliferation of OVCAR-3 and PANC-1 cancer cells, the latter were incubated with increasing concentrations of RM-133 for 72 h, and the IC_50_ of the drug then measured. RM-133 clearly had an antiproliferative activity on the tested cell lines, with IC_50_ values of 0.8 and 0.3 μM for OVCAR-3 and PANC-1, respectively (Fig 2). The IC_50_ values in the 0.1–1 μM range obtained here were similar to those previously measured in a panel of human cancer cell lines with this family of aminosteroids [23].

The OVCAR-3 cell line was derived from a patient refractory to cytotoxic chemotherapy, and is therefore an interesting model to investigate drug resistance [25]. The fact that RM-133 exhibited potent antiproliferative activity on a model (OVCAR-3) known for its chemoresistance, as well as on PANC-1 cells, prompted us to further investigate the *in vivo* activity of RM-133 in two tumor xenograft models using these two cancer cell lines.

### RM-133 inhibits growth of OVCAR-3 tumor xenografts

**Fig 3:**
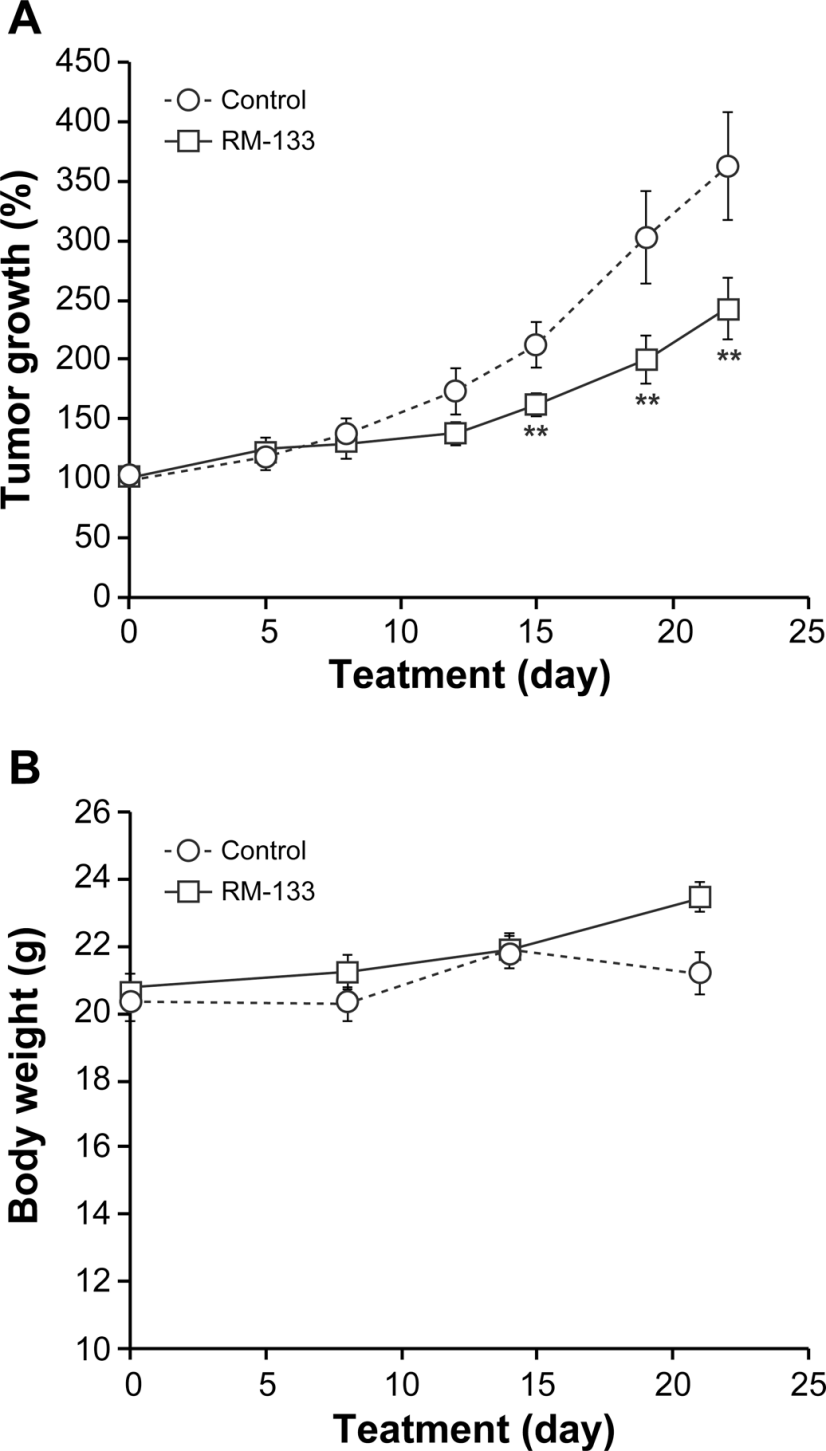
RM-133 in propylene glycol-based vehicle inhibits the growth of OVCAR-3 tumors xenografted in *nu/nu* nude mice. OVCAR-3 cells (5×10^6^ cells mixed with 30% matrigel) were inoculated s.c. into both flanks of mice. Mice bearing a tumor were injected once daily s.c. with RM-133 (0 or 60 mg/kg body weight) in 0.1 mL of propylene glycol:EtOH (92:8), for 21 days. Tumor size (A) and body weight of mice (B) were recorded. Data represent the mean ± SEM. **: RM 133-treated group is significantly different from control (P < 0.01).

Female nude mice were inoculated in both flanks with OVCAR-3 cells. Mice bearing tumors were randomized in 2 groups: one group was treated with RM-133 (60 mg/kg) and the other (control) received the vehicle only (propylene glycol:EtOH (92:8)). Tumors in the control group grew clearly more rapidly than those treated with the aminosteroid (Fig 3A). Starting from day 15 of drug therapy and until the end of the experimental period, RM-133-treated tumors became significantly smaller than in the control group, with a 60% difference between the two groups measured at the end of the treatment. Since the toxic potential of a given compound depends on its concentration and exposure time [37], and as mortality, clinical symptoms, and body weight changes are among the main indicators of its toxicity [38], it is noteworthy that over a 21-day treatment period with RM-133, there was no apparent effect on body weight (Fig 3B) nor apparent toxicity of the drug (e.g. abnormal behavior, death).

**Fig 4:**
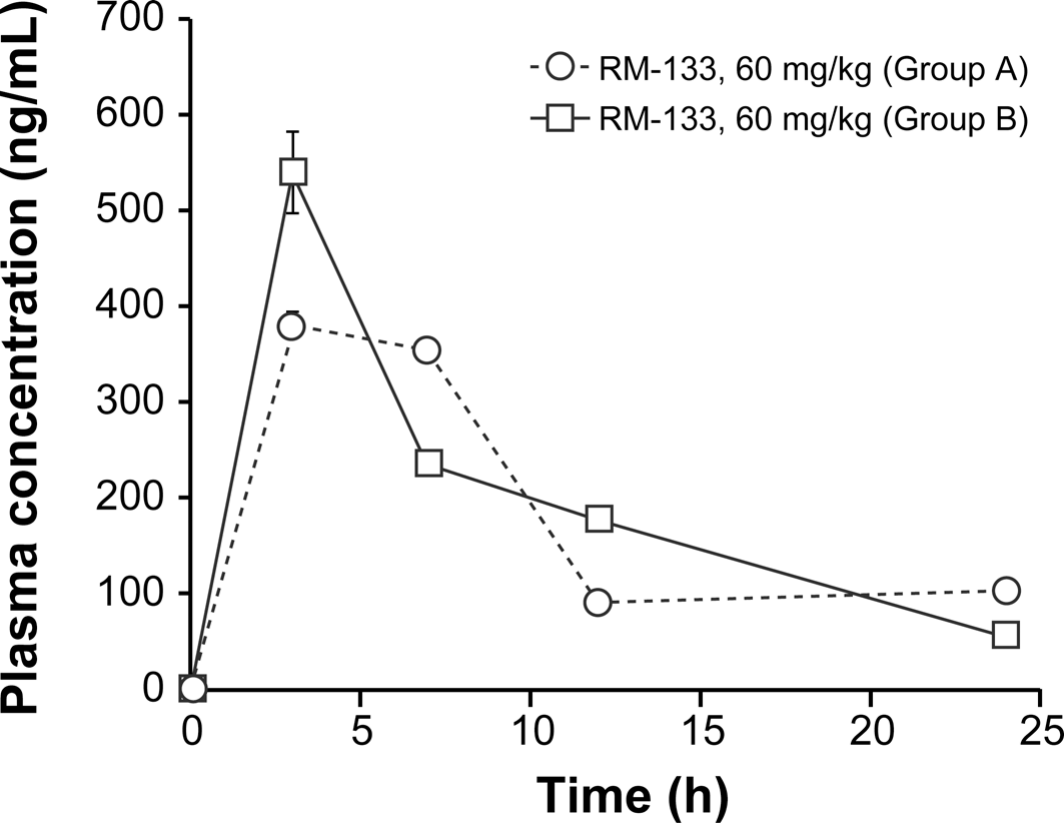
Time course of RM-133 plasma concentration following a single injection. RM-133 (60 mg/kg) in 0.1 mL of propylene glycol:EtOH (92:8) was injected s.c. into *nu/nu* nude mice. Group A: mice were previously treated daily for 21 days with RM-133 at 60 mg/kg; group B: mice not previously exposed to the drug.

At the end of the OVCAR-3 xenograft experiment, both control and RM-133-treated groups separately received a single s.c. dose of RM-133 (60 mg/kg). Blood was collected after 3, 7, 12, and 24 h, and the drug plasma concentration measured by LC-MS/MS. The time course of RM-133 plasma concentration was similar in groups A and B. The drug exhibited a classic drug-releasing profile [39], with a bolus delivery of RM-133 followed by a sustained decrease in its plasma concentration (Fig 4). The maximum average plasma concentration (460 ng/mL) was observed 3 h following the bolus injection, and had decreased to 81 ng/mL after 24 h. These results demonstrate that RM-133 does not accumulate in blood during the 21 days-xenograft experiment. Tumors of the mice treated with RM-133 were also collected at necropsy in order to measure the quantity of RM-133. This later was present inside the tumor at a concentration of 1.2 μM (773 ng/g), which corresponds roughly to the concentration of RM-133 that inhibits 50% of OVCAR-3 cell proliferation (IC_50_ = 0.8 μM). This result is in accordance with the 60% reduction of tumor size progression observed in the first OVCAR-3 xenograft experiment. Thus, RM-133 is not sufficiently concentrated inside the tumor to fully inhibit its growth.

### RM-133 plasma concentration exhibits a logarithmic dependence on the amount injected

**Fig 5:**
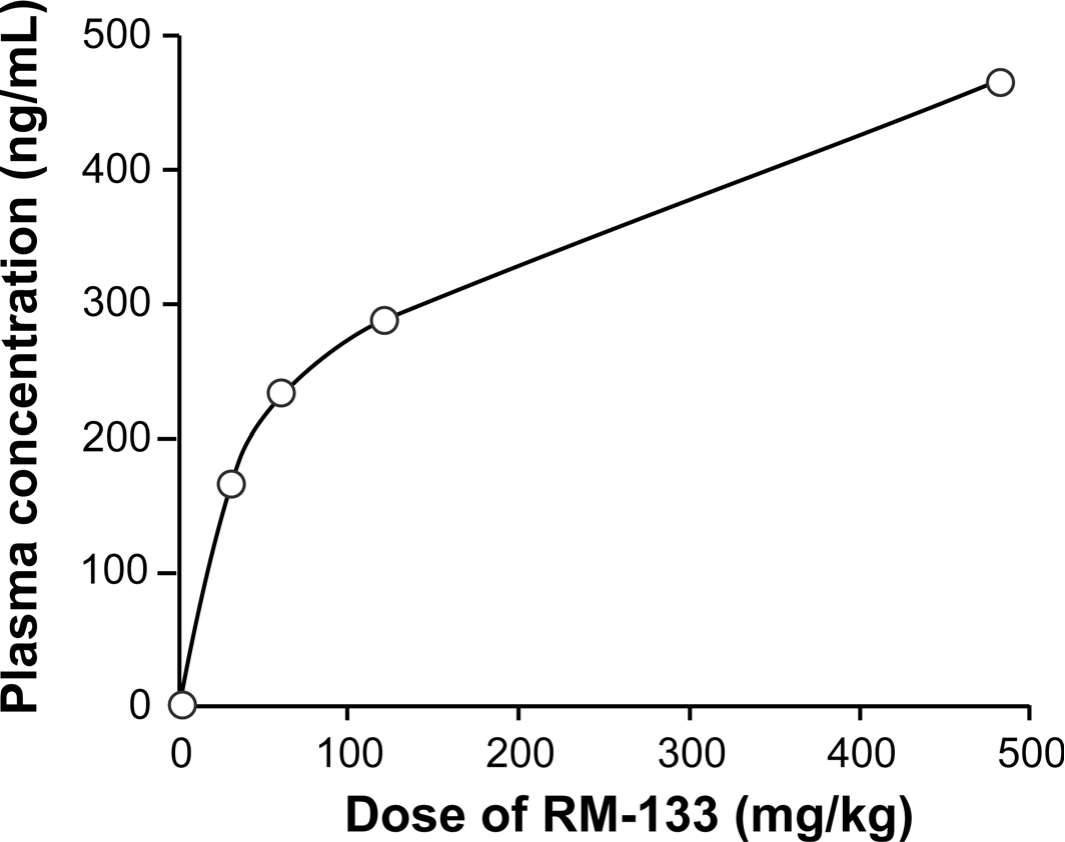
Plasma concentration of RM-133 as a function of the dose (30, 60, 120, and 480 mg/kg) injected. RM-133 was measured by LC-MS/MS 12 h following a single s.c. injection in Balb/c mice, using propylene glycol:EtOH (92:8) as vehicle. The volume of injection is 0.1 mL except for the highest dose used (480 mg/kg), where 0.2 mL were used. The volume of 0.2 mL, with and without RM-133, produced some motion discomfort.

In previous experiments, RM-133 had demonstrated a maximal antitumor activity of 57% against HL60 xenografts [32], which is comparable to the 60% inhibition value measured in this report towards OVCAR-3 xenografts. At the end of the OVCAR-3 xenograft experiment, RM-133 plasma concentration measured 12 h after a single bolus injection of 60 mg/kg (in 0.1 mL) in groups A and B (Fig 4) was 178 and 91 ng/mL, respectively. This result prompted us to examine the dose-plasma level relationship of a single s.c. injection of RM-133 using a range of different doses of the compound. As shown in Fig 5, the plasma concentration (167 ng/mL) reached after the injection of 30 mg/kg represents only 17% of the injected amount, and this percentage decreased further with higher doses of RM-133. Thus, the trend of the dose-level relationship suggests that increasing the RM-133 dose leads to a plateau, likely as a result of low plasma solubility or of the poor bioavailability of RM-133 in the vehicle used (propylene glycol:EtOH (92:8)). However, the dose of 480 mg/kg in 0.2 mL of vehicle was not tolerated by mice (Fig 5), which manifested with motion discomfort. Following these observations, the vehicle alone (0.2 mL of propylene glycol:EtOH (92:8)) was administered to the mice. As suspected, reactions were the same in the animals, indicating that the vehicle itself (propylene glycol:EtOH (92:8), at 0.2 mL) was the culprit. The maximum volume tolerated was next determined as 0.1 mL. These observations led us to investigate alternative vehicles that would allow to yield higher RM-133 plasma levels with minimal adverse effects.

### Vehicle optimization: screening of alternative vehicles for optimal RM-133 administration

Non-aqueous solvents such as dimethyl sulfoxide and polyethylene glycol, detergents (e.g. Tween, vegetable oils, etc.) and solubilizers (e.g. β-cyclodextrin, methylcellulose) offer strategic options for enhancing compound solubility in the drug discovery process [30, 39]. For this reason, 11 different vehicles previously used with steroidal derivatives were investigated. Firstly, based on the solubility of RM-133 in each vehicle (Table A in S1 File), eight vehicles were selected for a second round of investigation. In the latter experiments, mice received a single s.c. injection of each drug-free vehicle for tolerance and behavioral evaluation (Table B in S1 File). Based on our observations at different post-injection times (0.25–96 h), we eliminated the vehicle #3 (castor oil-based). In fact, following the administration of vehicle #3 alone, mice displayed many discomfort symptoms (itching, jumping, and microphtalmia), and during the following four days, mice presented wounds averaging ~24 mm^2^ at the site of injection.

In a third experiment, the seven best tolerated vehicles containing RM-133 were injected s.c. in mice (120 mg/kg) and blood samples collected after 3 and 12 h for RM-133 plasma concentration measurement (Table 1). The highest RM-133 plasma concentration reached after 3 h was obtained with vehicle #6, followed (in that order) by vehicles #5, #2, #1, #7, #4, and #8. As expected, RM-133 plasma concentration had decreased after 12 h, but vehicle #6 still yielded the highest levels after the latter interval. In contrast, vehicle #8 yielded the lowest plasma concentration at both time intervals. Based on the results obtained from the three experiments described above (Tables A and B in S1 File, Table 1), we concluded that (i) RM-133 was poorly soluble in vehicle #5 (small lumps), (ii) that vehicles #1 and #7 led to skin lesions and vehicle #8 yielded the lowest plasma concentrations of the analyzed set. On the other hand, vehicle #6 led to the highest plasma concentrations of the set, whereas vehicles #2 and #4 did not show any adverse effect on mice. We thus selected vehicles #2, #4, and #6 to perform an additional tolerance test.

**Table 1:** Time course of RM-133 plasma concentrations with various vehicles.

#	Vehicle	Plasma concentration	
		(ng/mL)^a^	
		3 h	12 h
1	Propylene glycol (92%)	314 ± 2	189 ± 5
	EtOH (8%)		
2	Aqueous 0.4% methylcellulose (92%)	449 ± 5	171 ± 10
	EtOH (8%)		
3	Castor oil (65%)	NT^b^	
	EtOH (10%)		
	Benzyl alcohol (10%)		
	Benzyl benzoate (15%)		
4	Sunflower oil (92%)	285 ± 5	173 ± 6
	EtOH (8%)		
5	Sunflower oil (92%)	642 ± 20	374 ± 9
	Tetrahydrofuran (8%)		
6	Aqueous 25% β-cyclodextrin (92%)	2963 ± 121	1783 ± 47
	EtOH (8%)		
7	Sesame oil (89.7%)	302 ± 7	214 ± 8
	EtOH (7.8%)		
	Benzyl benzoate (1%)		
	Benzyl alcohol (1%)		
	Tween 80 (0.5%)		
8	Soya oil (92%)	137 ± 2	52 ± 1
	EtOH (8%)		

^a^Single injection of RM-133 (120 mg/kg) in 0.1 mL of vehicle.

^b^NT: this vehicle was not tested in this experiment based on tolerance and behavioral evaluation in mice.

### Effect of repeated subcutaneous injections of RM-133 in 3 selected vehicles (tolerance test)

RM-133 was administered to mice every other day using vehicles #2, #4, and #6. Animals were treated for one week with vehicles #2 and #4, but only once with vehicle #6. Mice behavior was monitored during the experiment, and macroscopic observation of organs was performed at necropsy (Table C in S1 File). Ten hours following the s.c. injection of the first dose of RM-133 (120 mg/kg/0.1 mL) in aqueous 25% β-cyclodextrin:EtOH (92:8) (vehicle #6), mouse mobility was considerably reduced. Twenty-four hours later, their general condition had further deteriorated, as seen with seclusion, dehydration, microphthalmia, bristling, accelerated breathing rate, and abdomen distension. Based on these observations, we abruptly terminated experiment with vehicle #6, 24 h post-injection. At necropsy, all mice in that group showed hemorrhagic lungs, loaded stomachs along with empty intestines, and 75% exhibited slightly enlarged kidneys. When correlated with the mouse deaths previously noted when administering vehicle #6 alone, these observations clearly indicated that the cyclodextrin-based vehicle was not tolerated by mice, and vehicle #6 was therefore eliminated from our screening studies.

Interestingly, and in accordance with the observations from the experiment performed with vehicles only, no adverse effect was noted when mice were treated with RM-133 using vehicles #2 and #4. Because the oily vehicle #4 (sunflower oil:EtOH (92:8) generated blisters at the injection site, we limited treatment to a single injection of RM-133 (240 mg/kg/0.2 mL /every other day) for the xenograft experiments. However, the aqueous vehicle #2 (aqueous 0.4% methylcellulose:EtOH (92:8)) allowed to perform multiple injections (240 mg/kg/0.2 mL, BID/every other day) without adverse effect on mice. At necropsy, organs did not show any detectable abnormality. Thus, both vehicles #2 and #4 were included for comparison for the *in vivo* studies on RM-133 antitumor activity.

### Antitumor activity of RM-133 on OVCAR-3 xenografts using two different vehicles

**Fig 6:**
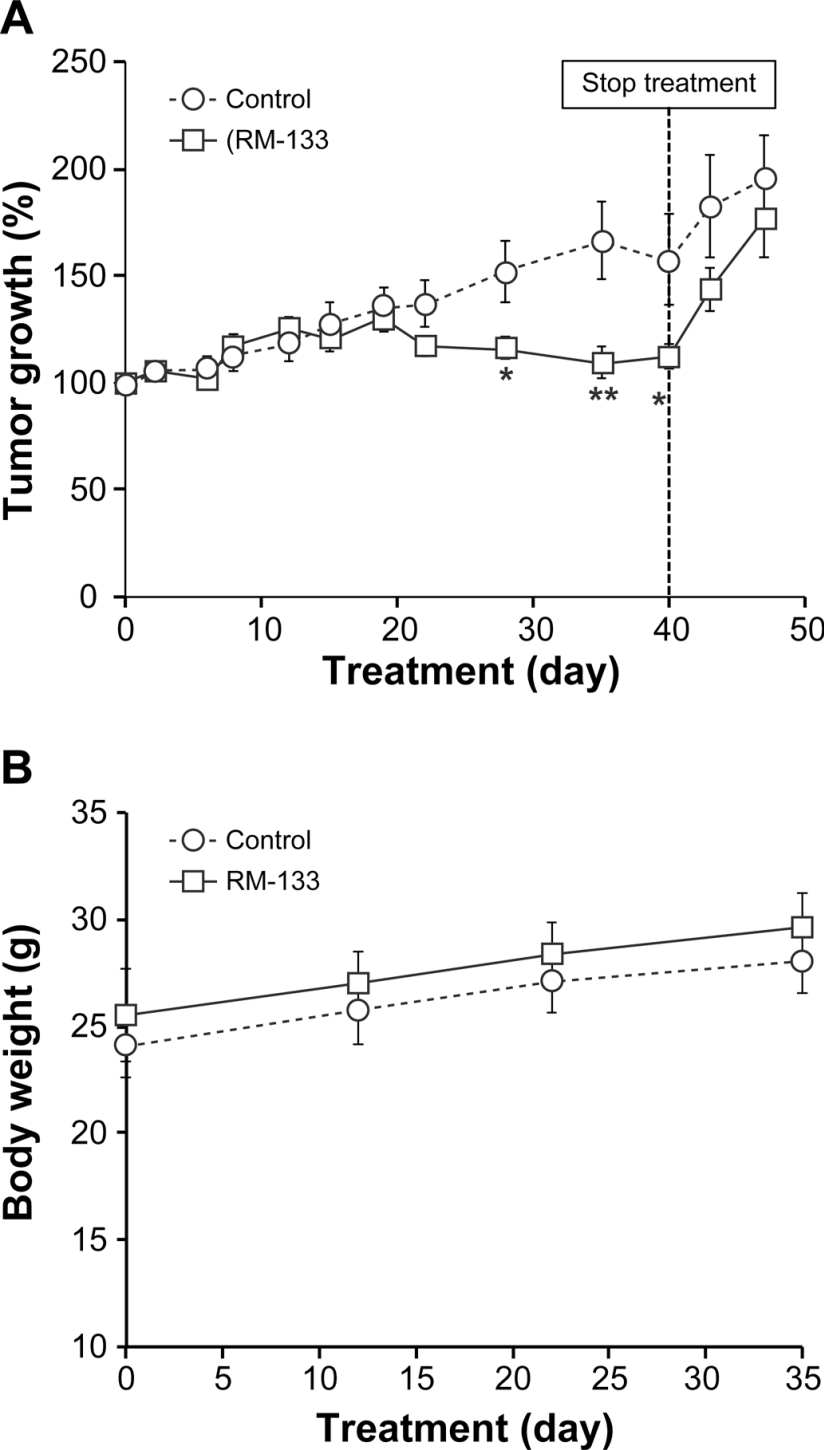
RM-133 in sunflower-based vehicle inhibits the growth of OVCAR-3 tumors xenografted in *nu/nu* nude mice. OVCAR-3 cells (5×10^6^ cells mixed with 30% matrigel) were inoculated s.c. into both flanks of mice. Tumor-bearing mice were injected s.c. with RM-133 (240 mg/kg body weight) or vehicle only (0.2 mL of sunflower oil:EtOH (92:8)) every other day. Tumor size (A) and body weight of mice (B) were recorded. Data represent the mean ± SEM **: RM-133-treated group is significantly different from control (P < 0.01). *: RM-133-treated group is significantly different from control (P < 0.05).

With the sunflower-based vehicle (Fig 6A), RM-133 started to inhibit the tumor growth after 20 days, but the effect became significant only at day 28. By the end of treatment (40 days), RM-133 had completely inhibited (i.e. by 100%) OVCAR-3 tumor growth. However, the oily nature of vehicle #4 generated big blisters, which also hampered tumor measurements. When RM-133 treatment was stopped at day 40 due to vehicle-induced effects, tumor growth resumed and no significant difference subsisted between the two groups seven days after treatment termination (Fig 6A). The latter results show that the aminosteroid RM-133 was responsible for tumor growth inhibition. No significant difference in body weight was observed between the untreated (control) and treated (RM-133) group (Fig 6B). Furthermore, no toxicity symptom was either observed during the study or at necropsy.

**Fig 7:**
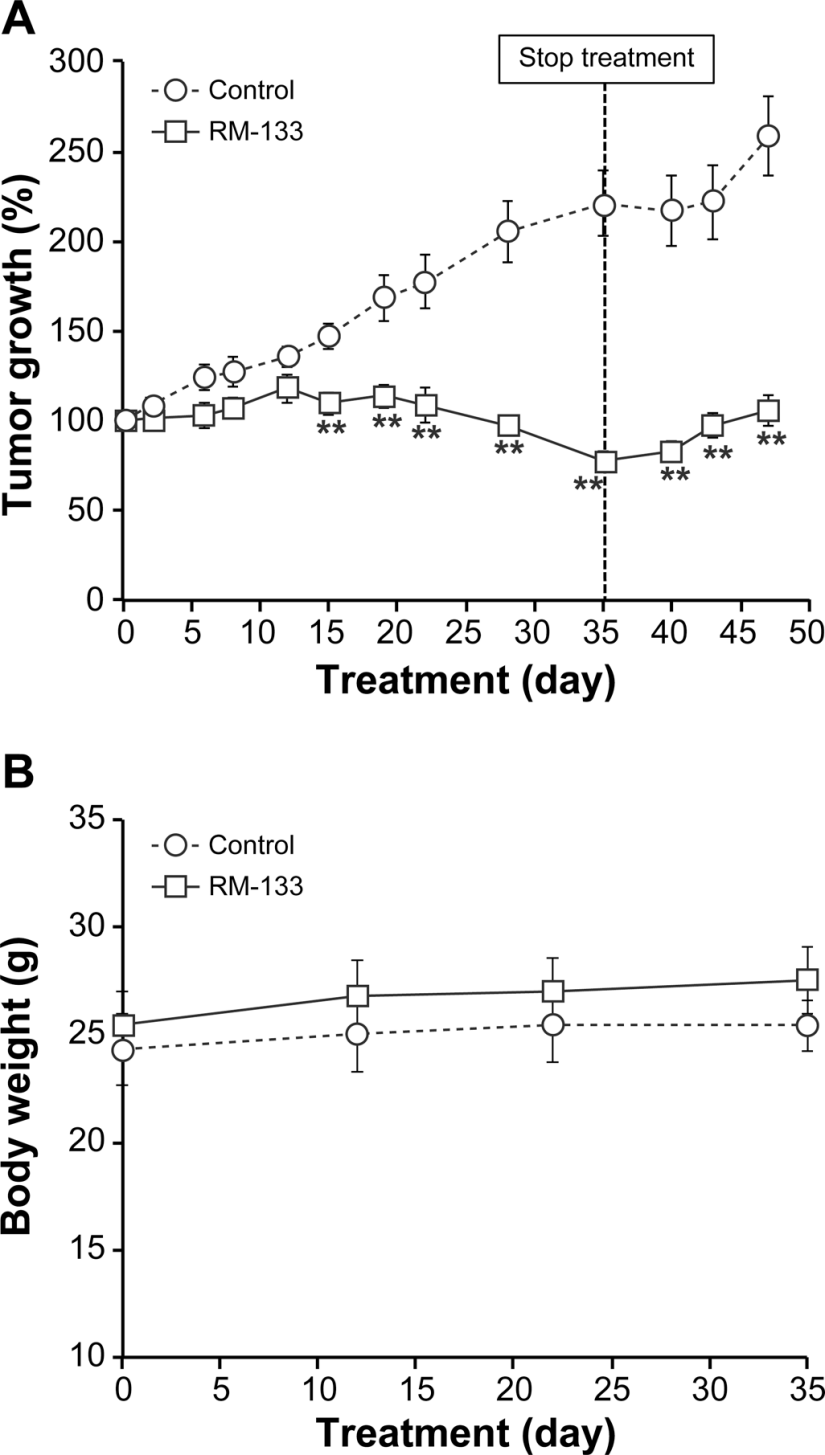
RM-133 in methylcellulose-based vehicle inhibits the growth of OVCAR-3 tumors xenografted in *nu/nu* nude mice. OVCAR-3 cells (5×10^6^ cells mixed with 30% matrigel) were inoculated s.c. into both flanks of mice. Tumor bearing mice were injected s.c. with RM-133 (240 mg/kg body weight) or vehicle only (0.2 mL of aqueous 0.4% methylcellulose:EtOH (92:8)) twice daily, every other day. Tumor size (A) and body weight of mice (B) were recorded. Data represent the mean ± SEM **: RM-133-treated group is significantly different from control (P < 0.01).

With the methylcellulose-based vehicle, tumor growth became significantly different between the treated and control groups as early as at day 15 (Fig 7A), and this difference persisted until the end of the experimental period (day 35). In fact, RM-133 treatment efficiently abrogated the rapid growth of OVCAR-3 tumors, whose surface area was decreased by the aminosteroid. After 28 days, RM-133 had completely inhibited (by 100%) tumor growth, and had further decreased tumor size to 78% of its initial value by the end of treatment (35 days). After treatment interruption, RM-133 still maintained its complete blockade of tumor progression until at least 12 days after the end of treatment (Fig 7A). Interestingly, neither abnormal behavior nor death was recorded over the 35-day treatment period with RM-133. Moreover, vehicle #2 did not affect body weight (Fig 7B) or lead to any overt toxicity symptom over the whole experimental period as well as at necropsy.

### Antitumor activity of RM-133 on PANC-1 xenografts

**Fig 8:**
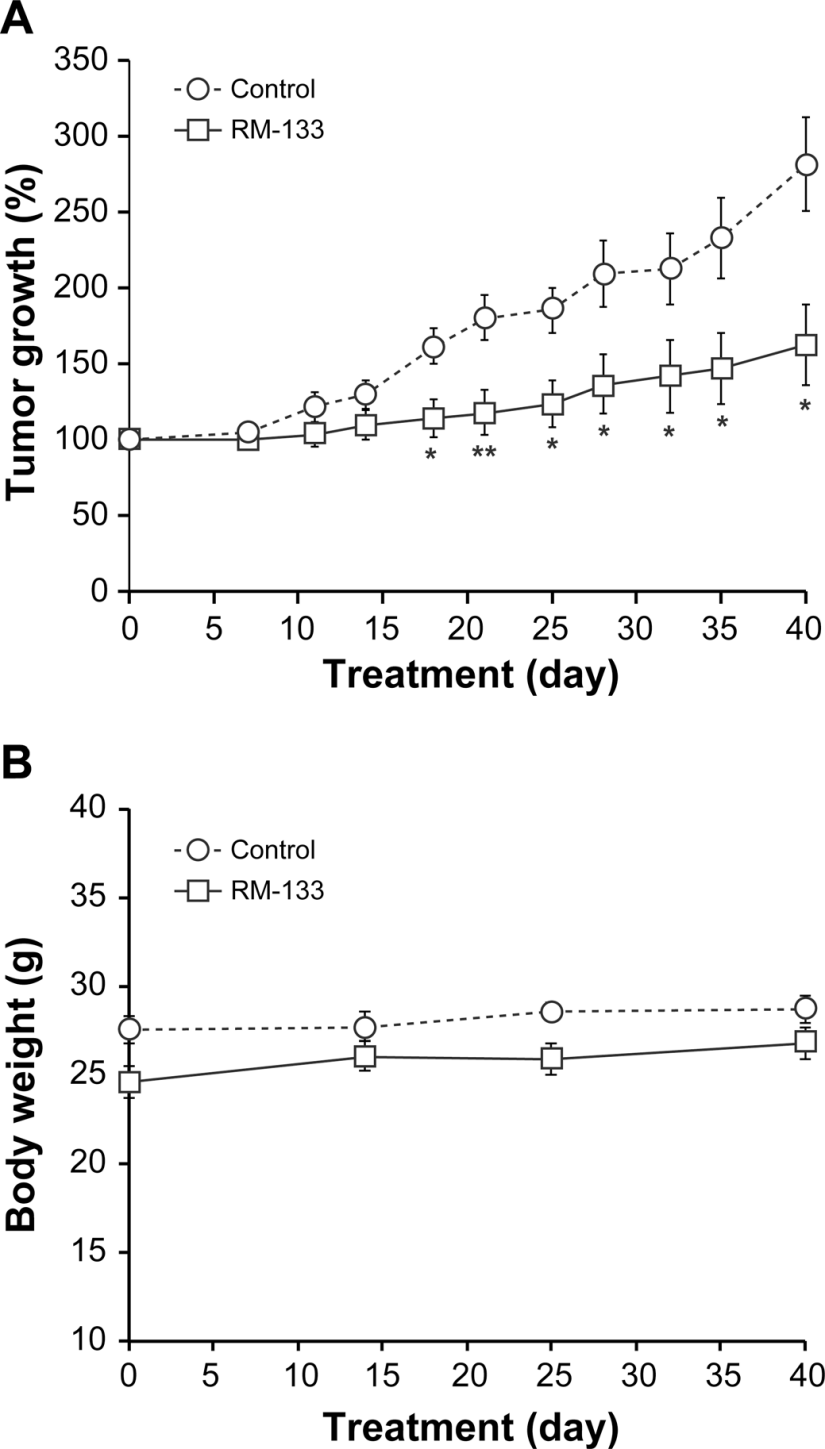
RM-133 in methylcellulose-based vehicle inhibits the growth of PANC-1 tumors xenografted in *nu/nu* nude mice. PANC-1 cells (5×10^6^ cells mixed with 30% matrigel) were inoculated s.c. into both flanks of mice. Tumor-bearing mice were injected s.c. with RM-133 (240 mg/kg body weight) or vehicle only (0.2 mL of aqueous 0.4% methylcellulose:EtOH (92:8)) twice daily, every other day. The tumor size (A) and body weight of mice (B) were recorded. Data represent the mean ± SEM. **: RM-133-treated group is significantly different from control (P < 0.01) *: RM-133-treated group is significantly different from control (P < 0.05).

As with ovarian cancer, pancreatic cancer typically shows resistance to currently available therapies and presents a very poor prognosis. Based on the results obtained with the OVCAR-3 xenografts, we used vehicle #2 (aqueous 0.4% methylcellulose:EtOH (92:8) to assess the effect of RM-133 on the PANC-1 pancreatic cancer tumor xenograft model. RM-133 progressively inhibited PANC-1 tumor growth until day 22, where a 75% reduction of tumor size progression was observed (Fig 8A). At the end of the treatment period (40 days), PANC-1 tumor progression was reduced by 63%, indicating that the aminosteroid possessed significant, albeit only partial antitumor activity towards the PANC-1 pancreatic tumor model. As already shown with the OVCAR-3 tumor xenograft experiments, body weight was not affected by treatment (Fig 8B), and no apparent toxicity could be detected either during the experimental period study or at necropsy.

## Conclusion

The cytotoxic activity of the aminosteroid RM-133 has been first evaluated *in vitro* in two human cancer cell lines, and the IC_50_ values in the low micromolar range that were obtained (0.8 μM for OVCAR-3 (ovary) and 0.3 μM for PANC-1 (pancreas)) prompted us to assess the potential of RM-133 in tumor xenograft models. RM-133 (60 mg/kg/day) reduced OVCAR-3 tumor xenograft growth by 60% in nude mice, using propylene glycol:EtOH (92:8) as vehicle and s.c. injections. The failure to achieve a stronger tumor growth inhibition pointed towards a poor bioavailability of RM-133 with the vehicle used. Upon investigating the solubility, plasma concentration, and tolerance of RM-133 delivered with 11 different vehicles, two alternative vehicles (aqueous 0.4% methylcellulose:EtOH (92:8) and sunflower oil:EtOH (92:8)) were selected for further xenograft experiments. Thus, long-term treatment with RM-133 reduced PANC-1 pancreatic tumor growth by 63% in methylcellulose-based vehicle. Interestingly, RM-133 completely inhibited OVCAR-3 tumor growth using either sunflower-based and methylcellulose vehicles; and using the latter vehicle only, RM-133 further reduced tumor size down to 78% of its initial size. However, the antitumor response observed using the oily, sunflower-based vehicle was delayed and less efficient compared to the methylcellulose-based vehicle. Furthermore, as observed with the methylcellulose-based vehicle, RM-133 maintained its antitumor action against the OVCAR-3 model, even one week after cessation of treatment. RM-133 was well tolerated by mice during the whole experimental period, and no weight loss was recorded. These promising results that have been obtained with ovarian and pancreatic cancer models, which are known as highly refractory to current therapies and as having a very poor prognosis, urge us to pursue our studies with the aminosteroid RM-133 and optimize its dosage, schedule, and mode of delivery, especially regarding its effect towards pancreatic cancer.

## Supporting Information

S1 File Table A. RM-133 solubility in 11 injection vehicles; Table B. Effect of a single s.c. injection of 8 vehicles in mice behavior; Table C. Effect of repeated s.c. injections of RM-133 using 3 preselected vehicles. (PDF)
