# Supplementary material for: The Aminosteroid Derivative RM-133 Shows In Vitro and In Vivo Antitumor Activity in Human Ovarian and Pancreatic Cancers
Source: PLoS One. 2015 Dec 14;10(12):e0144890. doi: 10.1371/journal.pone.0144890 (PMC4682813; doi:10.1371/journal.pone.0144890)
Supplement: S1 File — (PDF) [file pone.0144890.s001.pdf]

The aminosteroid derivative RM-133 shows in vitro and in vivo antitumor activity in human ovarian and pancreatic cancers

Lucie Carolle Kenmogne[1,2], Diana Ayan[1], Jenny Roy[1], René Maltais[1], Donald Poirier[1,2]*

[1] Laboratory of Medicinal Chemistry, Endocrinology and Nephrology Unit, CHU de Québec - Research Center, Québec (Québec), Canada

[2] Department of Molecular Medicine, Faculty of Medicine, Université Laval, Québec (Québec), Canada

* Corresponding author: E-mail: donald.poirier@crchul.ulaval.ca

Supporting Information

Table A. RM-133 solubility in 11 injection vehicles.

Table B. Effect of a single s.c. injection of 8 vehicles in mice behavior.

Table C. Effect of repeated s.c. injections of RM-133 using 3 preselected vehicles.

Table A. RM-133 solubility in 11 injection vehicles.

#	Vehicles	Appearance Vehicle alone	Appearance Vehicle + RM-133 (27.6 mg/mL)
1	Propylene glycol (92%)<br>EtOH (8%)	Clear<br>Transparent<br>Colorless	Clear<br>Transparent<br>Colorless
2	Aqueous 0.4% methylcellulose (92%)<br>EtOH (8%)	Clear<br>Transparent<br>Colorless	Opaque<br>Milky<br>Whitish<br>Small lumps
3	Castor oil (65%)<br>EtOH (10%)<br>Benzyl alcohol (10%)<br>Benzyl benzoate (15%)	Clear<br>Transparent<br>Yellowish<br>Viscous +++	Clear<br>Transparent<br>Yellowish ++<br>Viscous +++
4	Sunflower oil (92%)<br>EtOH (8%)	Clear<br>Transparent<br>Yellowish	Hazy +<br>Yellowish +<br>Viscous +
5	Sunflower oil (92%)<br>Tetrahydrofuran (8%)	Clear<br>Transparent<br>Yellowish<br>Viscous	Hazy ++<br>Yellowish +<br>Small lumps<br>Viscous ++
6	Aqueous 25% β-cyclodextrin (92%)<br>EtOH (8%)	Opaque<br>Milky<br>Whitish<br>2 phases	Opaque<br>Milky<br>Whitish<br>Homogeneous
7	Sesame oil (89.7%)<br>EtOH (7.8%)<br>Benzyl benzoate (1%)<br>Benzyl alcohol (1%)<br>Tween 80 (0.5%)	Clear<br>Transparent<br>Yellowish +++<br>Viscous+	Hazy +<br>Transparent<br>Yellowish +++<br>Viscous +
8	Soya oil (92%)<br>EtOH (8%)	Clear<br>Transparent<br>Yellowish+<br>Viscous +	Hazy +<br>Transparent<br>Yellowish +<br>Viscous +
9	RPMI without FBS (92%)<br>EtOH (8%)	Clear<br>Transparent	Whitish<br>Lumps +++
10	Saline qsp (89.5%)<br>DMSO (10%)<br>Tween 80 (0.5%)	Clear<br>Transparent	Whitish<br>Lumps +++
11	Saline qsp (98.2%)<br>Benzyl alcohol (0.9%)<br>Tween 80 (0.4%)<br>Carboxymethylcellulose (0.5%)	Clear<br>Transparent	Whitish<br>Lumps ++++

Table B. Effect of a single subcutaneous injection of 8 vehicles (0.1 mL) in mice behavior.[a]

#	Vehicle	Time of observation (h)								
		0.25	1	3	7	23	26	28	30	96
1	Propylene glycol (92%)<br>EtOH (8%)	Oily hair (grade 3), active and normal mice	Well-groomed mice, less oily hair (grade 1), active mice	Well-groomed and normal mice	Well-groomed and normal mice	Normal hair, active and normal mice	Normal hair, active and normal mice	Normal hair, active and normal mice	Normal hair, active and normal mice	#3: no wound<br>#13: 5-mm lesion<br>#82: 3-mm lesion<br>#91: 10-mm opened wound
2	Aqueous 0.4% methylcellulose (92%)<br>EtOH (8%)	Normal hair, active and normal mice	Normal hair, active and normal mice	Normal hair, active and normal mice	Normal hair, active and normal mice	Normal hair, active and normal mice	Normal hair, active and normal mice	Normal hair, active and normal mice	Normal hair, active and normal mice	Normal hair, no wound
3	Castor oil (65%)<br>EtOH (10%)<br>Benzyl alcohol (10%)<br>Benzyl benzoate (15%)	Oily hair (grade 8), overactive mice, bumps at the injection site	Overactive mice, moving by leaps and bounds, scratching, do not groom, very oily hair (grade 8)	Glued hair (grade 8), moving by leaps and bounds, ached back, semi-closed eyes, overactive mice	Glued hair (grade 8), moving by leaps and bounds, ached back, semi-closed eyes, mice do not cluster to rest	Well-groomed mice, less oily hair (grade 4), active and normal mice	Well-groomed mice, less oily hair (grade 4), active and normal mice	Well-groomed mice, less oily hair (grade 3), active and normal mice	Almost normal hair (grade 1-2), active and normal mice	#12: 4-mm wound<br>#21: 6-mm wound<br>#79: 7-mm mange and 4-mm wound<br>#86: 5-mm and 3-mm wounds
4	Sunflower oil (92%)<br>EtOH (8%)	Oily hair (grade 3), active and normal mice	Well-groomed mice, (grade 3), active mice	Active and normal mice, oily hair (grade 4)	Active and normal mice, oily hair (grade 4)	Active and normal mice, less oily hair (grade 2)	Active and normal mice, almost normal hair (grade 1)	Active and normal mice, almost normal hair (grade 1)	Normal hair, active and normal mice	Normal hair, no wound

5	Sunflower oil (92%) Tetrahydrofuran (8%)	Oily hair (grade 4), active and normal mice	Well-groomed mice, (grade 4), active mice	Active and normal mice, oily hair (grade 4)	Active and normal mice, oily hair (grade 4)	Active and normal mice, less oily hair (grade 2)	Active and normal mice, almost normal hair (grade 1)	Active and normal mice, almost normal hair (grade 1)	Normal hair, active and normal mice	Normal hair, no wound
6	Aqueous 25% β-cyclodextrine (92%) EtOH (8%)	Normal hair, active and normal mice	Normal hair, active and normal mice	Normal hair, active and normal mice	Normal hair, active and normal mice	Normal hair, active and normal mice, one mice found dead	Normal hair, active and normal mice	ormal hair, active and normal mice	Normal hair, active and normal mice	#17: 8-mm lesion<br>#25: 8-mm lesion<br>#76: no wound<br>#20: dead
7	Sesame oil (89.7%) EtOH (7.8%) Benzyl benzoate (1%) Benzyl alcohol (1%) Tween 80 (0.5%)	Oily hair (grade 5), active and normal mice	Oily hair (grade 5), active and normal mice	Active and normal mice, oily hair (grade 5)	Active and normal mice, oily hair (grade 5)	Active and normal mice, less oily hair (grade 2)	Active and normal mice, almost normal hair (grade 1)	Active and normal mice, almost normal hair (grade 1)	Normal hair, active and normal mice	#15: 3-mm and 10-mm wounds<br>#18: No wound<br>#84: 2-mm lesion<br>#87: no wound
8	Soya oil (92%) EtOH (8%)	Oily hair (grade 6), active and normal mice	Well-groomed mice, oily hair (grade 5), active and normal mice	Active and normal mice, oily hair (grade 4)	Active and normal mice, oily hair (grade 4)	Active and normal mice, less oily hair (grade 2)	Active and normal mice, almost normal hair (grade 1)	Active and normal mice, almost normal hair (grade 1)	Normal hair, active and normal mice	#5: No wound<br>#9: No wound<br>#24: No wound<br>#81: 3-mm lesion

[a]Oily hair grades: Grade 0: normal mouse - Grade 10: drop of topical oil.

Table C. Effect of repeated subcutaneous injections of RM-133 using 3 preselected vehicles.[a]

Vehicle	#	Dose of RM-133	Observations (days)							
			1 (Injection)	2	3 (Injection)	4	5 (Injection)	6	7 (Injection)	8 (Necropsy)
Aqueous 0.4% methylcellulose (92%) EtOH (8%)	2	2 x 240 mg/kg/0.2 mL<br>(AM and PM)	Normal	Normal	Normal	Normal	Normal	Normal	Normal	3/3: white deposit at the injection site<br>Normal organs
Sunflower oil (92%) EtOH (8%)	4	2 x 120 mg/kg/0.1 mL<br>(AM and PM)	Oily hair following injection. Grade 4	AM oily hair (grade 1)<br>PM normal hair	Oily hair following injection. Grade 4	AM oily hair (grade 1)<br>PM normal hair	Oily hair following injection. Grade 4	AM oily hair (grade 1)<br>PM normal hair	Oily hair following injection. Grade 4	AM: Oily Hair AM (grade 1)<br>3/3: light white deposit at the injection site<br>3/3: oil blisters<br>3/3: slightly marbled and less dense spleen
Aqueous 25% β-cyclo-dextrin (92%) EtOH (8%)	6	1 x 120 mg/kg/0.1 mL<br>(AM)	10 h-following injection<br>Reduced mobility<br>Undone nest	Isolated and dehydrated mice<br>Microphthalmia<br>Bristling hair<br>Accelerated breathing rate<br>Distended abdomen<br>Necropsy<br>4/4: hemorrhagic lungs<br>4/4: loaded stomach but empty bowels<br>3/4: slightly enlarged kidneys<br>1/4 White deposit at the injection site	-	-	-	-	-	-

[a]Oily hair grades: Grade 0: normal mouse - Grade 10: drop of topical oil.
